# The complete chloroplast genome of the newly recorded species *Tainia acuminata* Averyanov (Orchidaceae) from China

**DOI:** 10.1080/23802359.2022.2077666

**Published:** 2022-05-26

**Authors:** Xia-lian Ou, Ying Qin, Li-guo Zhang, Ting-guang Sun, Xin-mei Qin

**Affiliations:** a College of Life Science, Guangxi Normal University, Guilin, China; b Guangxi Key Laboratory of Plant Conservation and Restoration Ecology in Karst Terrain, Guangxi Institute of Botany, Guangxi Zhuang Autonomous Region and Chinese Academy of Sciences, Guilin, China; c Jiangxi Province Key Laboratory of Watershed Ecosystem Change and Biodiversity, Institute of Life Science and School of Life Sciences, Nanchang University, Nanchang, China; d Department of Medicine, Guangxi University of Science and Technology, Liuzhou, China

**Keywords:** Chloroplast genome, *Tainia acuminata*, phylogenetic analysis

## Abstract

The complete chloroplast genome sequence of *Tainia acuminata* Averyanov was assembled and the phylogenetic relationship of the species to other taxa in Subtrib. Bletlinae was inferred in this study. The length of the complete chloroplast sequence is 157,603 bp, and it contains a large single-copy (LSC) region of 86,336 bp, a small single-copy (SSC) region of 18,129 bp, and two inverted repeat (IRA and IRB) regions of 26,569 bp. A total of 134 genes were annotated including 89 protein-coding genes, 37 tRNA, and eight rRNA. Phylogenetic analysis showed that *T. acuminata* was closely related to *T. cordifolia*, and the genus was closely related to a clade consisting of *Calanthe*, *Phaius*, and *Cephalantheropsis*.

*Tainia acuminata* Averyanov 2013, a plant of Orchidaceae, is distributed in China and Vietnam (Averyanov 2013; Yuan et al. 2020). It has high ornamental value owing to its characteristic leaf shape and large beautiful flowers. *T. acuminata* has not been sequenced yet according to our knowledge, and the phylogenetic placement, species identification, genetic diversity, and conservation situation, etc. of the species remain unknown. The chloroplast sequences or genomes have been widely used in the studies of these areas (Daniell et al. 2016; Li et al. 2021). Here, we report the complete chloroplast genome of *T. acuminata* and its phylogenetic position in Subtrib. Bletlinae.

A sample of *T. acuminata* was collected from Jiuwanshan National Nature Reserve in Guangxi, China (25°11′26″ N, 108°47′31″ E), and the voucher specimen was deposited at the Herbarium of Guangxi Institute of Botany (http://www.gxib.cn/spIBK/, contact person: Chun-Rui Lin, Email: chunruilin@tom.com) under the voucher number QY20190410027. The total DNA was extracted from silica-gel dried leaves by the CTAB method (Doyle and Doyle 1987). The genomic paired ends (PE150) sequencing was performed on NovaSeq 6000 (in Novogenecorp, Tianjin, China). Approximately, 1.6 Gb of clean data were gained after quality filtering using fastp (Chen et al. 2018). The complete chloroplast genome was de novo assembled using GetOrganelle (Jin et al. 2020), and annotated using PGA (Qu et al. 2019) and the online tool GeSeq (Tillich et al. 2017) with reference to the chloroplast genome of *T. dunnii* (NC_045862). The sequence was submitted to the GenBank (accession number: OL489753).

The complete chloroplast genome of *T. acuminata* has a length of 157,603 bp, consisting of a large single-copy (LSC 86,336 bp) region, a small single-copy (SSC 18,129 bp) region, and two inverted repeat (IRA and IRB 26,569 bp) regions. The overall GC content of the complete chloroplast genome is 37.3%. It contains 134 genes, including 89 protein-coding genes, 37 tRNA, and eight rRNA.

To investigate the phylogenetic position of *T. acuminata*, maximum-likelihood (ML) phylogenetic tree was reconstructed using RAxML (Stamatakis 2014) based on 17 chloroplast genomes using *Eria lasiopetala* as the outgroup. The phylogenetic tree indicated that *T. acuminata* was most closely related to *T. cordifolia* (BS = 100%) (Figure 1), which was consistent with morphological observation (Averyanov 2013; Yuan et al. 2020). The genus was most closely related to a clade consisting of *Calanthe*, *Phaius*, and *Cephalantheropsis*.

**Figure 1.**
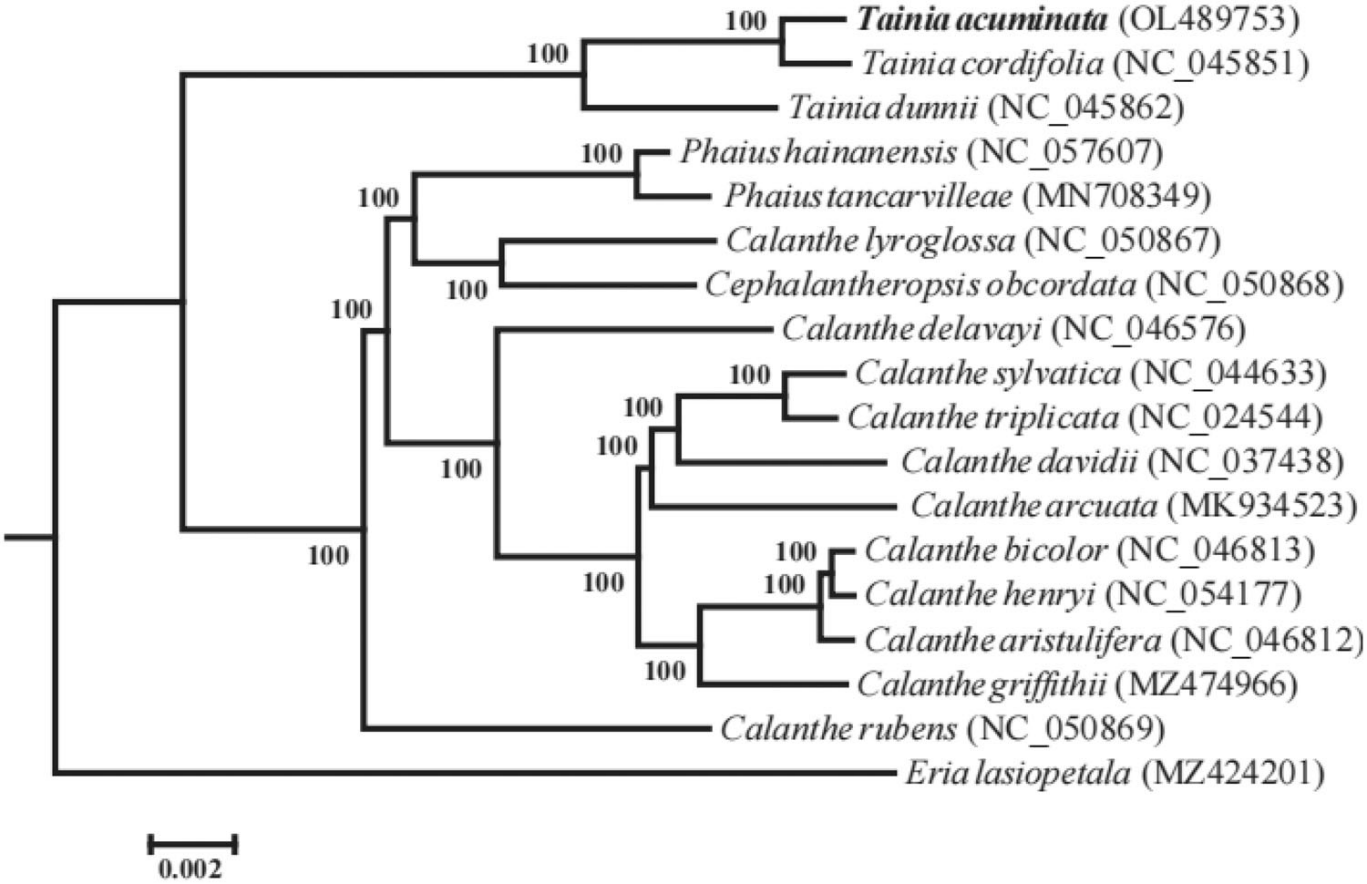
ML phylogenetic tree of 17 species of Subtrib. Bletlinae based on chloroplast genome sequences. Bootstrap support values (1000 replicates) are shown at the nodes.

## Data Availability

The genome sequence data that support the findings of this study are openly available in GenBank of NCBI at https://www.ncbi.nlm.nih.gov/ under the accession no. OL489753. The associated BioProject, SRA, and BioSample numbers are PRJNA781090, SRR16964329, and SAMN23235068, respectively.
